# 

**Published:** 1900-03-10

**Authors:** 


					rntti axjarirjLij. " The standarcldf highest purity.'^? Lancet. March 10th, 1900.
CADBURY'S
CADc??.RY'S * H
COCOA
ABSOLUTELY PURE. ^ ^ NO DRUGS OR ALKALIES USED.
ascoit Western INF'mMAR*
No. 702.\y0l. XXVII. [SfiJ5-a Saturday,
March 10, 1900.
[Subscription Terms,] pRICE TWOPENCE.

				

